# The effect of anticoagulants in blood collection tubes on *Plasmodium falciparum* transmission in direct membrane feeding assays

**DOI:** 10.1093/trstmh/trab095

**Published:** 2021-06-28

**Authors:** Wouter Graumans, Kjerstin Lanke, Geert-Jan van Gemert, Manon Alkema, Marga van de Vegte-Bolmer, Teun Bousema, Katharine A Collins

**Affiliations:** Radboud University Medical Center , Radboud Institute for Health Sciences , Department of Medical Microbiology, 268, PO Box 9101, 6500 HB Nijmegen, The Netherlands; Radboud University Medical Center , Radboud Institute for Health Sciences , Department of Medical Microbiology, 268, PO Box 9101, 6500 HB Nijmegen, The Netherlands; Radboud University Medical Center , Radboud Institute for Health Sciences , Department of Medical Microbiology, 268, PO Box 9101, 6500 HB Nijmegen, The Netherlands; Radboud University Medical Center , Radboud Institute for Health Sciences , Department of Medical Microbiology, 268, PO Box 9101, 6500 HB Nijmegen, The Netherlands; Radboud University Medical Center , Radboud Institute for Health Sciences , Department of Medical Microbiology, 268, PO Box 9101, 6500 HB Nijmegen, The Netherlands; Radboud University Medical Center , Radboud Institute for Health Sciences , Department of Medical Microbiology, 268, PO Box 9101, 6500 HB Nijmegen, The Netherlands; Department of Immunology and Infection, London School of Hygiene and Tropical Medicine, Keppel St, London WC1E 7HT, UK; Radboud University Medical Center , Radboud Institute for Health Sciences , Department of Medical Microbiology, 268, PO Box 9101, 6500 HB Nijmegen, The Netherlands

**Keywords:** anticoagulant, DMFA, gametocyte, heparin, malaria, transmission

## Abstract

**Background:**

Direct membrane feeding assays assess the transmission potential of malaria-infected individuals using whole blood collected in anticoagulant vacutainers.

**Methods:**

The potential inhibitory effect of four commonly used anticoagulants on gametocyte infectivity to mosquitoes was assessed in standard membrane feeding assays with cultured *Plasmodium falciparum*.

**Results:**

Infection burden in mosquitoes was significantly reduced when blood was collected in sodium citrate and EDTA. Transmission was highest when blood was collected in lithium heparin and sodium heparin, although a concentration-dependent inhibition of mosquito infection was also observed.

**Conclusions:**

Although anticoagulants can reduce transmission efficiency, lithium heparin and sodium heparin are the best anticoagulants for evaluating malaria transmission.

## Introduction

Malaria is transmitted to female *Anopheles* mosquitoes when they take a blood meal from an infected human and ingest gametocytes, the transmissible form of the malaria parasite. Mosquito feeding assays are used to determine the transmission potential of gametocyte carriers to identify reservoirs of malaria and determine their contribution to onward transmission. Direct skin feeding assays allow mosquitoes to feed directly on the skin of infected individuals. These assays closely mimic natural blood feeding behavior but are not widely used, in part because of the discomfort associated with repeated assessments and ethical challenges in vulnerable populations. Direct membrane feeding assays (DMFAs) instead offer venous collected whole anticoagulated blood to mosquitoes via membrane feeders, and are more commonly used. Phlebotomy minimizes discomfort and also permits logistical flexibility—when and where feeds are performed—but has technical challenges. Skin feeding assays have been shown to give higher mosquito infection rates compared with DMFAs,^1^ fueling hypotheses that either gametocytes sequester in capillaries in the skin^2^ or that technical factors in DMFAs affect transmission efficiency. Anticoagulants used during blood collection for DMFAs could potentially have differential effects on transmission efficiency.^3,4^ A better understanding of the impact of anticoagulants is therefore important for optimizing DMFAs and for interpreting DMFA results in the context of natural malaria transmission. Here, the four most commonly used vacutainer anticoagulants, lithium heparin (LiHep), sodium heparin (NaHep), sodium citrate or EDTA, were evaluated in standard membrane feeding assays (SMFAs), where cultured gametocytes were offered to mosquitoes via a membrane feeder, to compare their impact on *Plasmodium falciparum* transmission.

## Materials and Methods

Venous blood samples were collected in BD Vacutainers (Becton, Dickinson and Company, Plymouth, UK) with anticoagulant additives: LiHep (ref. 368496), NaHep (ref. 367869), sodium citrate (ref. 363048) and EDTA (ref. 367525), then directly inverted after blood collection. Heparin dilutions were prepared in human AB serum (universal recipient) with either LiHep salt (Sigma-Aldrich, Saint Louis, Missouri, United States, H0878-100KU) or NaHep salt (Sigma-Aldrich, H3393-10KU) and added to blood collected in vacutainers without anticoagulant (ref. 368500). *Plasmodium falciparum* strain NF135 was cultured in an automated tipper system.^5^ Infected *Anopheles stephensi* mosquitoes (Sind-Kasur strain) were kept at 26°C and 80% humidity, with a reversed 12-/12-h day/night cycle. For each SMFA 300 µl of freshly drawn blood was kept at 37°C, added to 30 µl of cultured gametocytes and fed by mini-feeders to a total of 60 mosquitoes (2 cups of 30 per condition). Mosquitoes were dissected 7 d later and midguts stained with 1% mercurochrome for quantification of oocysts by microscopy. Differences in oocyst intensity between conditions (anticoagulants) and in relation to anticoagulant concentration were examined by negative binomial regression models, incorporating a random effect for replicate cups and experiments.

## Results and Discussion

**Figure 1.**
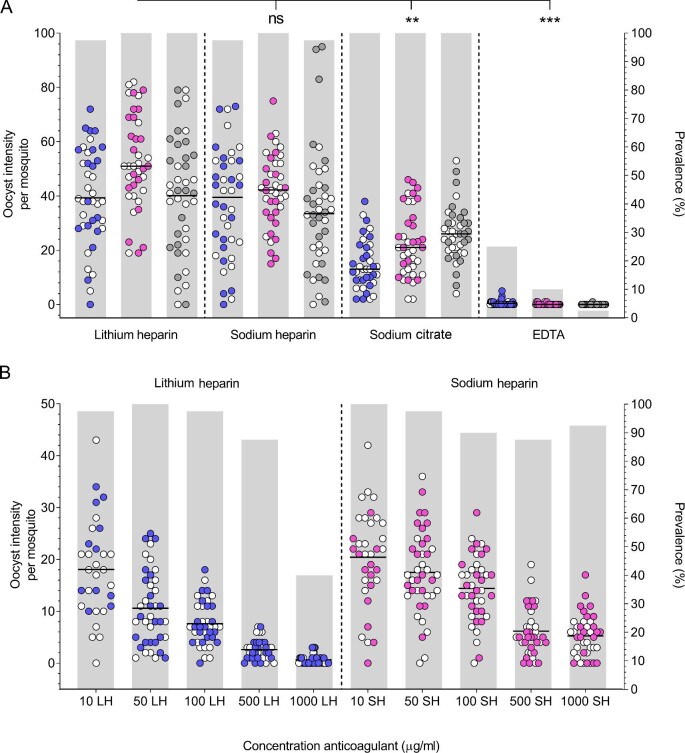
Effect of anticoagulants in vacutainers on *Plasmodium falciparum* transmission. (A) Venous blood samples with anticoagulant additives were added to cultured *P. falciparum* gametocytes and fed to 2 cups of 30 mosquitoes per condition. Data from duplicate cups are shown pooled (open and closed circles), and three independent replicate experiments were performed (shown in pink, blue and gray). (B). The effect of heparin was further explored at a range of lithium heparin (LH) and sodium heparin (SH) concentrations. Gametocytes were mixed with whole blood containing the desired anticoagulant concentration and fed to 2 cups of 30 mosquitoes. Data from duplicate cups are shown pooled (open and closed circles). **P ≤ 0.01, ***P ≤ 0.001.

Oocyst intensity was significantly lower in SMFAs using sodium citrate (2.1-fold, p=0.007) and EDTA anticoagulants (261.6-fold, p<0.001) compared with LiHep, but oocyst intensity was similar between LiHep and NaHep (p=0.605) (Figure 1A). The different effects of anticoagulants on transmission may be explained by their mechanisms of action to prevent clotting. Heparin, a sulfated polysaccharide, inhibits activation of thrombin, thereby blocking the production of the clotting protein fibrin, whereas sodium citrate and EDTA are chelators of ionized calcium, an essential metal required for the coagulation cascade. Calcium may be involved in the process of male gametocyte activation and ookinete motility.^4^ Chelating this ion may contribute to the observed effect of sodium citrate and EDTA on transmission.^3,4^

Whilst LiHep and NaHep had the least impact on transmission of the anticoagulants tested, they may still reduce transmission efficiency compared with blood samples without anticoagulants (as used in skin feeding). Therefore, we evaluated the effect of heparin in SMFAs at increasing concentrations and observed a statistically significant trend for reduced oocyst density with increasing concentration of LiHep (p<0.001) and NaHep (p<0.001) (Figure 1B). This effect was seen with both NaHep and LiHep at concentrations similar to those present in standard heparin vacutainers (NaHep ∼91 µg/ml and LiHep ∼88 µg/ml). This demonstrates that heparin can interfere with the transmissibility of *P. falciparum* gametocytes and may contribute to the difference observed between DMFAs and skin feeding assays. A similar effect of heparin inhibiting transmission to mosquito vectors has been observed with another malaria parasite, *Plasmodium berghei*, although at higher concentrations.^3^ There, heparin was found to inhibit ookinete transition to the basal side of the midgut and prevent development into oocysts.

### Conclusion

We examined four of the most commonly used anticoagulants and found that EDTA and sodium citrate are associated with lower gametocyte infectivity to mosquitoes. Our findings further indicate that the preferred anticoagulants for mosquito feeding experiments, NaHep and LiHep, may reduce transmission efficiency at concentrations similar to those present in standard vacutainer tubes. This suggests that a partial reduction of infectivity due to anticoagulants may explain differences between direct skin feeding experiments and DMFAs and warns against partially filling tubes as this will increase the concentration of these anticoagulants further.

## Data Availability

The datasets used and/or analyzed during the present study are available from the corresponding author upon request.
